# Epigenetic and Transcriptional Control of Erythropoiesis

**DOI:** 10.3389/fgene.2022.805265

**Published:** 2022-03-07

**Authors:** Maeve Wells, Laurie Steiner

**Affiliations:** Department of Pediatrics, University of Rochester, Rochester, NY, United States

**Keywords:** erythroid, epigenetic, transcription, anemia, disease

## Abstract

Erythropoiesis is a process of enormous magnitude, with the average person generating two to three million red cells every second. Erythroid progenitors start as large cells with large nuclei, and over the course of three to four cell divisions they undergo a dramatic decrease in cell size accompanied by profound nuclear condensation, which culminates in enucleation. As maturing erythroblasts are undergoing these dramatic phenotypic changes, they accumulate hemoglobin and express high levels of other erythroid-specific genes, while silencing much of the non-erythroid transcriptome. These phenotypic and gene expression changes are associated with distinct changes in the chromatin landscape, and require close coordination between transcription factors and epigenetic regulators, as well as precise regulation of RNA polymerase II activity. Disruption of these processes are associated with inherited anemias and myelodysplastic syndromes. Here, we review the epigenetic mechanisms that govern terminal erythroid maturation, and their role in human disease.

## Introduction

**FIGURE 1:**
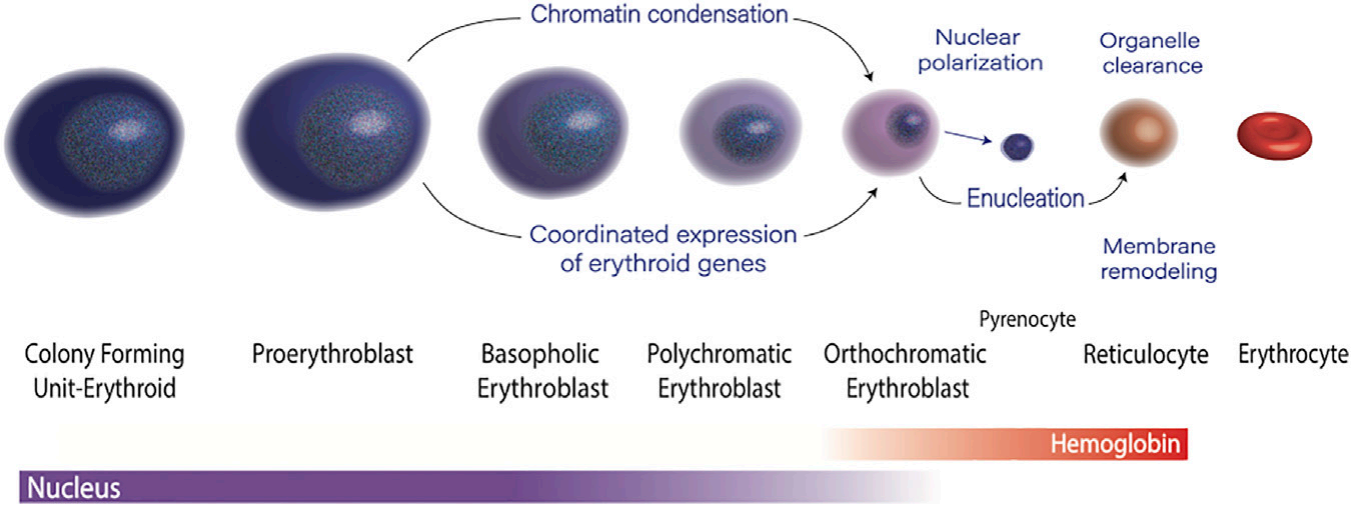
Overview of Erythropoiesis.

Erythropoiesis is the process of making red blood cells. At each stage of maturation, erythroblasts are both phenotypically distinct and have a unique transcriptomic profile (An et al., 2014) and chromatin landscape. The morphologic changes include a progressively acidophilic appearance due to accumulation of hemoglobin, a steady decrease in cell size, and dramatic nuclear condensation, which culminates in enucleation. This process requires the coordinated effort of epigenetic regulators and transcription factors, as well as precise regulation of RNA polymerase II activity (Figure 1). The average human makes two to three million red cells per second to maintain steady state, and avoid anemia (Palis 2014). Not surprisingly, many people cannot maintain this impressive output, and anemia affects nearly 1/3 of the global population.(Kassebaum et al., 2014). Defects in terminal erythroid maturation, such as nuclear condensation defects, or asynchronous maturation of the nucleus and cytoplasm, are commonly found in myelodysplastic syndromes, and inherited anemias. Understanding the molecular mechanisms that govern terminal erythroid maturation is essential to understanding the how mutations or other genetic perturbations result in dyserythropoiesis, and to designing rational therapies. In this review we will discuss the epigenetic and transcriptomic control of erythropoiesis, with a focus on how disruption of these fundamental processes contributes to human disease.

## The Chromatin Landscape Is Dynamic in Maturing Erythroblasts

During the maturation of a committed progenitor to a reticulocyte, the nucleus condenses to ∼1/10th of its original volume prior to enucleation.(Ji et al., 2011). This dramatic change in nuclear phenotype is associated with dynamic changes in the in the epigenetic landscape. Each stage of erythropoiesis is associated with a unique pattern of chromatin accessibility, (Schulz et al., 2019), with maturation-specific enhancers driving stage-specific gene expression profiles (Ludwig et al., 2019). The establishment of appropriate patterns of 3D chromatin architecture is essential for this process.(Oudelaar et al., 2020). In addition, at the onset of terminal maturation, erythroblasts undergo a dramatic, genome wide decline in DNA methylation, (Shearstone et al., 2011; Yu et al., 2013), and factors that control DNA methylation, particularly the TET family of proteins, are important for regulating erythropoiesis.(Yan et al., 2017). In eukaryotes, all DNA is bound by histone proteins into chromatin. Posttranslational modifications of N-terminal “tails” of these proteins are key regulators of chromatin structure and gene expression. Changes in histone modifications during erythroid maturation have been implicated as regulators of gene expression and nuclear condensation.(Zhao et al., 2016b).

Acetylation of histone tails is generally associated with open chromatin structure and transcriptional activation, and regulation of histone acetylation is critical for the establishment appropriate patterns of erythroid gene expression and chromatin structure. Studies of friend leukemia transformed erythroblasts suggested that global deacetylation of histone residues is necessary for erythroid chromatin condensation.(Popova et al., 2009). Mass spectrometry studies in cultured human erythroblasts demonstrated terminal erythroid maturation was associated with loss of specific histone modifications, most notably loss of H4K16 acetylation, but not with a global decline in histone acetylation levels.(Murphy et al., 2021). Modifications of the histone H4 tail are particularly critical determinates of higher order chromatin structure. They directly impact the ability of neighboring nucleosomes to interact and form compact chromatin structures, with H4K16 acetylation disrupting tight interactions between neighboring nucleosomes and promoting a relaxed chromatin architecture.(Shogren-Knaak et al., 2006; Zhang et al., 2017). In addition, modifications to the H4 tail are important for recruiting epigenetic readers that further regulate chromatin structure and gene expression, (Lee and Workman 2007; Lamonica et al., 2011; Gong et al., 2016), and are involved in the regulation of RNA polymerase II pausing.(Kapoor-Vazirani et al., 2011).

Although a global decline in histone acetylation does not accompany human erythropoiesis, deacetylation of select histone modifications likely plays an important role. Several histone deacetylases (HDACs), including HDACs 2, 3, and five are highly expressed in maturing erythroblasts and have essential roles in erythropoiesis.(Ji et al., 2010). HDAC5, in particular is upregulated in late stage erythroblasts. Chemical inhibition of HDAC5 leads to elevated levels of H4 acetylation, which is accompanied by disrupted chromatin condensation and impaired enucleation.(Wang et al., 2021). HDAC2 has also been shown to have an essential role in chromatin condensation during terminal erythroid maturation.(Ji et al., 2010). Importantly, the essential role for some HDACs during erythroid maturation is in part due to their action non-histone substrates, such as the protein mDia2, which has an essential role in cytokinesis and enucleation.(Li et al., 2017).

Dynamic changes in histone methylation also occur during erythropoiesis, with both histone methyltransferases, such as NSD1 (Tauchmann et al., 2020) and SETD8, (Malik et al., 2015; Malik et al., 2017; Myers et al., 2020; DeVilbiss et al., 2015), and histone demethylases, such as LSD1, (Kerenyi et al., 2013; Yu et al., 2021), playing critical role in erythroid gene regulation and erythroid maturation. The distribution of well-characterized histone markers of activation and repression, including H3K4me3, H3K27me3, and H3K9me3 appear to be established at the time of erythroid commitment, and are relatively stable during terminal maturation.(Wong et al., 2011; Wu et al., 2011). In contrast, histone H4 Lysine 20 mono-methylation (H4K20me1), which facilities intra-nucleosomal interactions and chromatin compaction accumulates dramatically in late-stage erythroblasts prior to enucleation. Setd8 is the sole histone methyltransferase that mono-methylates H4K20me1.(Oda et al., 2009). Although Setd8 is expressed broadly in proliferating tissues, it is highly expressed in erythroid cells compared to most other cell types.(Wu et al., 2016; Myers et al., 2020). Loss of Setd8, results in defective chromatin condensation and disruption of the nuclear membrane.(Malik et al., 2017). Setd8 deficient cells also had a significant disruption of erythroid gene expression. They were able to express erythroid-specific genes, such as the globins, but were unable to repress the non-erythroid transcriptome, accompanied by an increase in chromatin accessibility at those loci. (Myers et al., 2020).

Histone marks that reflect active transcription through gene bodies, such as H3K36me2/3 and H3K72me2 also vary dynamically during terminal maturation, (Murphy et al., 2021), and play a critical role in the regulation of erythroid gene expression. Chromatin immunoprecipitation experiments demonstrated that changes in H3K79me2, in terminally maturing erythroblasts correlates closely with changes in gene expression.(Wong et al., 2011). Deletion of DOT1L, the H3K79 histone methyltransferase, is embryonic lethal due to dramatic anemia from failure of primitive erythropoiesis. Underlying this anemia was abnormal expression of both GATA2 and PU.1, transcription factors critical for cell fate decisions during hematopoiesis.(Feng et al., 2010). ASH1L, a histone methyltransferase with H3K4 and H3K36 methyltransferase activity, also plays an important role in erythropoiesis, and has been implicated as a regulator of beta globin, with a point mutation in ASH1L associated with a beta thalassemia phenotype.(Breton et al., 2016). In late stage erythroblasts, histone marks that reflect active transcription, including H3K36me3 and H3K79me2, decrease dramatically, likely reflecting the general decline in active RNA polymerase II that occurs during terminal erythroid maturation.(Murphy et al., 2021).

## Control of Transcription Plays a Central Role in Terminal Erythroid Maturation

Transcription is intimately linked to the chromatin landscape. Interactions between transcription factors and epigenetic modifications regulate the activity of RNA polymerase II, and in turn, active transcription by RNA polymerase II shapes the chromatin landscape.(Smolle and Workman 2013). As noted above, multiple studies suggest that the chromatin landscape for typical repressive histone marks are established at the time of erythroid commitment, and do not vary dramatically during maturation. (Wong et al., 2011; Wu et al., 2011). This suggests that control of transcription has a central role in the regulation of gene expression in maturing erythroblasts. The “transcription cycle” consists of transcription initiation, elongation, and termination, and gene expression can be regulated though control of any of the steps in the transcription cycle. *In vivo* deletion of factors that regulate the transcription cycle including HEXIM1, TIF1γ, TFIIs, JMJD6 and TRIM28, results in severe anemia, (Bose et al., 2004; Kunisaki et al., 2004; Ito et al., 2006; Montano et al., 2008; Bai et al., 2013; Hosoya et al., 2013), highlighting the importance of transcriptional control in erythropoiesis.

One of the best described mechanisms of transcriptional regulation is promoter-proximal pausing which allows dynamic changes in gene expression by controlling the release of promoter-bound, initiated RNA Polymerase II into active elongation.(Core and Lis 2008). At genes regulated by RNA Polymerase II pausing, double strand inducing factor (DSIF), and negative elongation factor (NELF) promote a “pause” after RNA Polymerase transcribes 30-60bp, with additional signals required for transition into active elongation. Pausing is a critical checkpoint in gene expression, as RNA Polymerase II cannot transition into active elongation without being phosphorylated by pTEFb, a complex of cyclinT1 (CCNT1) and cyclin dependent kinase 9 (CDK9).(Paparidis et al., 2017; Li et al., 2018). pTEFb can associate with tissue specific transcription factors, including GATA1 (Elagib et al., 2013) to facilitate RNA Polymerase II pause release at specific loci. Alternatively, pTEFb can also exist in a small nucleoprotein complex (the 7SK snRNP complex) where it interacts with the hexamethylene acetamide (HMBA) inducible protein HEXIM1, rendering it inactive, but capable of being targeted to specific loci.(Tan et al., 2016). HEXIM1 plays an essential role in terminal erythroid maturation. Alterations of HEXIM1 levels or function in erythroblasts leads to changes in the level of elongation competent RNA polymerase II, and disrupted terminal maturation with impaired expression of erythroid specific genes, such as those involved in heme synthesis. (Murphy et al., 2021).

Both the function and level of RNA polymerase II are tightly controlled in maturing erythroblasts. Terminal erythroid maturation is associated with a dramatic decline in the levels of both total and elongation competent RNA polymerase II, and changes in the occupancy of RNA polymerase II are highly associated with changes in erythroid gene expression. (Murphy et al., 2021). In contrast to most terminally maturing somatic cells, which retain their nuclei and therefore must have robust mechanisms for long term transcriptional silencing, maturing erythroblasts are destined to eject their nuclei following three to four cell divisions. Erythroblasts therefore have no need to expend cellular resources establishing large heterochromatin domains to maintain transcriptional repression. RNA Polymerase II “pausing” may actually be a misnomer in this population of cells, as many genes that appear to “pause” in intermediate erythroblasts never resume active transcription prior to enucleation.

Other steps in the transcription cycle are also essential for erythropoiesis. A recent paper using novel methods to assess nascent transcription during the terminal maturation of murine erythroblasts found that transcriptional initiation is a critical regulatory step in the transcription of many erythroid genes, and they utilized elegant functional studies to demonstrate that recruitment of RNA polymerase II is an important point of transcriptional regulation for the alpha and beta globin loci.(Larke et al., 2021). As levels of RNA polymerase II decline dramatically during erythroid maturation, allocation of RNA polymerase II to erythroid genes is a logical regulatory step in establishing appropriate patterns of gene expression during terminal maturation. In the future, it will be important to further delineate the mechanisms by which control of RNA polymerase II activity regulates erythroid gene expression.

## Cross Talk Between Epigenetic Modifiers, Transcription Factors, and the Transcription Machinery Is Essential for Terminal Erythroid Maturation

Cross talk between transcriptional regulators and epigenetic regulators are critical for establishing appropriate patterns of chromatin architecture and erythroid gene expression. GATA1, a master regulator of the erythroid transcriptional program, interacts extensively with epigenetic modifiers to shape the chromatin landscape. It can exist in both activating complexes, where it interacts with the histone acetyl transferase p300, (Blobel et al., 1998; Su et al., 2013; Zheng et al., 2014), and in repressive complexes where it interacts with members of the NuRD chromatin remodeling complex, including HDAC1 and HDAC2.(Rodriguez et al., 2005). The interplay between GATA1 and histone acetylation has been carefully dissected at the beta globin locus, where GATA1-mediated recruitment of CBP and p300 to enhancers is important for the establishment of tissue-specific histone acetylation patterns and higher order chromatin structure that facilitate high levels of globin expression in maturing erythroblasts.(Blobel et al., 1998; Kiekhaefer et al., 2002; Letting et al., 2003; Kim et al., 2020). Given the large excess of co-repressors compared to co-activators in the erythroid nucleus, (Gillespie et al., 2020), recruitment of activators such as HATs to target genes is likely a key mechanism by which GATA1 facilitates erythroid gene expression.

GATA1 can also alter patterns of histone methylation, and can work with the polycomb two repressive complex (PRC2 Complex) to shape the H3K27me3 landscape.(Ross et al., 2012). GATA factors also impact the composition of the PRC2 complex, with the GATA1/GATA2 switch promoting a developmental transition from expression of the histone methyltransferase EZH2 to the highly related histone methyltransferases EZH1. Following this developmental switch, EZH1 forms a non-canonical PRC2 complex with SUZ12, which positively affects gene expression.(Xu et al., 2015). Highlighting the role of GATA1 in shaping the epigenome, N-terminal truncating mutations of GATA1, termed GATA1s, fail to establish normal patterns of H3K27me3 during erythroid differentiation.(Ling et al., 2019). Consistent with an important role for transcription initiation in erythroid cells, GATA1 has been shown to interact directly with the basal transcription factor TAF10 which is important for the establishment of the erythroid transcriptional program, with erythroid deletion of TAF10 resulting in severe embryonic anemia.(Papadopoulos et al., 2015).

KLF1 is essential for both chromatin condensation and enucleation during terminal erythropoiesis.(Gnanapragasam et al., 2016; Gnanapragasam and Bieker, 2017). KLF1 promotes the expression of may genes important for chromatin condensation, including Setd8.(Tallack et al., 2010). The nan mutation of KLF1 has also been shown to impair chromatin condensation, in part due to dysregulated expression of the histone transporter XPO7, (Cantu et al., 2019), which is important for chromatin condensation during terminal erythroid maturation.(Hattangadi et al., 2014). KLF1 can interact with both HAT and HDAC complexes, (Chen and Bieker, 2004), and is also important for the recruitment of RNA polymerase II (Vinjamur et al., 2016) to the beta globin loci. Loss of KLF also leads to cell cycle perturbations and decreased expression of E2F2, (Pilon et al., 2008), which is an important cell cycle regulator that also has a role in driving chromatin condensation and facilitating enucleation in maturing erythroblasts.(Swartz et al., 2017). Similar to KLF1, TAL1 facilitates the expression of genes such as E2F2 that facilitate chromatin condensation during terminal erythroid maturation.(Kassouf et al., 2010). TAL1 can also shape the histone landscape by interactions with epigenetic modifiers such as p300. (Huang et al., 1999).

Other transcription factors also play a role in regulating the erythroid epigenome and chromatin architecture. For example, the transcription factor FOXO3 is upregulated during the late-stage of terminal erythroid maturation and promotes the expression of genes essential for terminal erythroid maturation, with loss of FOXO3 leading to multilobed nuclei and deficient enucleation (Liang et al., 2015). The transcription factor MYC also plays a key role in shaping the epigenome by promoting the expression of the histone acetyltransferase GCN5, and down regulation of MYC is necessary for chromatin condensation during terminal erythroid maturation. (Jayapal et al., 2010). In addition, the transcriptional regulator MAZ (MYC-associated zinc finger) occupies the alpha globin promoter and regulates erythroid maturation (Deen et al., 2021) and the transcription factor IKAROS contributes to gamma globin repression through recruitment of HDAC1 to the gamma globin promoter. (Bottardi et al., 2009).It is also important to note that epigenetic modifiers can regulate the expression of transcription factors. In maturing erythroblasts, Setd8 plays an important role in establishing appropriate patterns of gene expression, (DeVilbiss et al., 2013), and represses GATA2, and other key transcriptional regulators during erythroid differentiation.(DeVilbiss et al., 2015; Malik et al., 2015; Myers et al., 2020).

## Disruption of Erythroid Gene Regulation Contributes to Both Inherited and Acquired Anemias

Mutations of transcription factors and epigenetic regulators can lead to both inherited and acquired erythroid disorders. In addition, Genome Wide Association Studies have demonstrated that genetic variants associated with red cell phenotypes often occur in enhancers or other regulatory elements critical for the proper expression of the erythroid transcriptome.(van der Harst and Xhang, 2012; Su et al., 2013; Ulirsch et al., 2016; Choudhuri et al., 2020). Table 1 represents summary of transcription factors and epigenetic regulators that have been associated with inherited anemias.

**TABLE 1:** Transcriptional and epigenetic regulators associated with inherited anemias.

Factor	Function	Associated Disease(s)	References
GATA1	Transcription Factor	Mutations in GATA1	Campbell et al. (2013)
		• x-linked thrombocytopenia with beta thalassemia-like anemia	Balduini et al. (2004)
		• Thrombocytopenia with a congenital erythropoietic porphyria phenotype	Di Pierro et al. (2015)
		• Diamond Blackfan-like Anemia	Phillips et al. (2007)
		Mutations in GATA1 Binding Sites	Hollanda et al. (2006)
		• Congenital Dyserythropoietic Anemia Type II	Sankaran et al. (2012)
		• X-linked sideroblastic anemia	Klar et al. (2014)
		• congenital erythropoietic porphyria	Ciovacco et al. (2008)
		• pyruvate kinase deficiency	Russo et al. (2017)
			Kaneko et al. (2014)
			Wakabayashi et al. (2016)
KLF1	Transcription Factor	• Hydrops fetalis	Magor et al. (2015)
		• Congenital Erythropoietic Anemia Type IV	Lee et al. (2016)
		• Hereditary Persistence of Fetal Hemoglobin	Jaffray et al. (2013)
			Ravindranath et al. (2018)
			Borg et al. (2010)
RUNX1	Transcription Factor	• Familial platelet disorder with high risk of hematologic malignancy	Simon et al. (2020)
GATA2	Transcription Factor	• Multilineage dysplasia	Ganapathi et al. (2015)
		• Familial myelodysplastic syndrome	McReynolds et al. (2018)
		• Bone marrow failure with high risk of hematologic malignancy	Holland et al. (2018)
ATRX	Chromatin Remodeler	• X-linked Alpha Thalassemia Intellectual Disability	He et al. (2017)
ASH1L	Histone Methyltransferase	• Beta Thalassemia-like phenotype	Breton et al. (2016)
Codanin1 (CDAN1)	Implicated in chromatin assembly, but function incompletely understood	• Congenital Dyserythropoietic Anemia Type 1	Dgany et al. (2002)
SEC23B	Vesicle Formation	• Congenital Dyserythropoietic Anemia Type 2	Schwarz et al. (2009)
			Iolascon et al. (2010)

Mutations in GATA1, or GATA1-target sequences, cause anemia, which can be accompanied by thrombocytopenia, consistent with its role both megakaryopoiesis and erythropoiesis. Highlighting the essential nature of the cross talk that occurs between transcription factors and epigenetic modifiers, mutations that impair the ability of GATA1 to interact with co-activator and co-repressor complexes can lead to severe clinical phenotypes. For example the R216Q mutation, which impairs the ability of GATA1 to recruit the TAL1 activating complex, (Campbell et al., 2013), leads to x-linked thrombocytopenia with a beta thalassemia-like anemia (Balduini et al., 2004), while the closely related R216W mutation leads to anemia, thrombocytopenia, and a congenital erythropoietic porphyria phenotype.(Phillips et al., 2007; Di Pierro et al., 2015). Mutations of GATA1 that impair its ability to interact with the co-repressor FOG1 (Friend of GATA1) also lead to variable degrees of thrombocytopenia and anemia.(Nichols et al., 2000).

Mutations in GATA1-binding sites that lead impaired expression of the target gene have been linked with a number of hematologic disorders, (Ciovacco et al., 2008), including Congenital Dyserythropoietic Anemia Type II(Russo et al., 2017), X-linked sideroblastic anemia, (Kaneko et al., 2014; Wakabayashi et al., 2016), congenital erythropoietic porphyria, (Solis et al., 2001), and pyruvate kinase deficiency.(Wakabayashi et al., 2016). Next generation sequencing technologies, including gene panels, whole exome, and whole genome sequencing, are being more routinely incorporated into clinical use. The use of these technologies are likely to increase the detection of these types of pathologic variants and provide further insights into the role of GATA1, and other transcription factors, in inherited anemias.

Decreased GATA1 protein levels, have also been associated with human disease. DBA is an inherited erythroid failure syndrome that is associated with short stature and predisposition to cancer, most commonly caused by mutations in the ribosomal protein genes.(Tyagi et al., 2020). In DBA, haploinsufficiency of ribosomal protein genes can lead to decreased translation of the GATA1 protein, which then contributes to ineffective erythropoiesis.(Ludwig et al., 2014). Germline N-terminal truncating mutations of GATA1, GATA1s, result in an inherited erythroid failure that resembles DBA.(Hollanda et al., 2006; Sankaran et al., 2012; Klar et al., 2014). As noted above, the GATA1s mutation has an impaired ability to appropriately shape the epigenome during erythroid differentiation, leading to aberrant patterns of H3K27me3, and altered patterns of gene expression, (Ling et al., 2019), although the precise interactions are disrupted by the GATA1s mutation are incompletely understood.

KLF1 is also an essential erythroid transcription factor. (Nuez et al., 1995; Perkins et al., 1995). Mutations in KLF1 can also cause several different inherited erythroid disorders, including fetal anemia severe enough to cause hydrops fetalis, (Magor et al., 2015; Lee et al., 2016), congenital erythropoietic anemia type IV, (Jaffray et al., 2013; Ravindranath et al., 2018), and hereditary persistence of fetal hemoglobin (Borg et al., 2010). Intriguingly, studies of the Neonatal Anemia (nan) mutation in the mouse have shown that it alters the binding specificity of the KLF1 protein, resulting in disruption of the erythroid transcriptome.(Siatecka et al., 2010; Planutis et al., 2017). Germline mutations in other transcription factors, including GATA2, and RUNX1 can also contribute to inherited anemias. These transcription factors are also important in multipotent hematopoietic progenitors, and germline mutations in these factors are often associated with multilineage dysplasia, development of myelodysplastic syndrome, and increased risk of leukemia. (Ganapathi et al., 2015; McReynolds et al., 2018; Simon et al., 2020).

Mutations in epigenetic regulators have also been associated with inherited anemias. As noted above, a point mutation of the histone methyltransferase ASH1L has been associated with a beta thalassemia-like phenotype.(Breton et al., 2016). Mutations in the transcriptional regulator ATRX are associated with X-linked Alpha Thalassemia Intellectual Disability, (He et al., 2017), which occurs almost exclusively in males in and is associated with characteristic facial features, neurodevelopmental delays, genital urinary abnormalities, and anemia.(Stevenson 1993). ATRX is a chromatin remodeler that interacts with EZH2 (Cardoso et al., 1998) and is important for histone H3.3 incorporation (Goldberg et al., 2010). Mutations in ATRX lead to significant disruption of the epigenome, particularly DNA methylation pattern (Schenkel et al., 2017). Congenital Dyserythropoietic Anemia type I (CDA-I), is an autosomal recessive disease that presents with erythroid hyperplasia in the bone marrow. The erythroblasts in patients with CDA-I are frequently binucleate, have chromatin bridging, and most notably defective chromatin condensation. When visualized under electron microscopy, the chromatin of CDA-1 erythroblasts has a characteristic “spongy” or “swiss cheese” appearance” (Iolascon et al., 2020). CDA-1 is most commonly caused by mutations in Codanin-1 (CDAN1), (Dgany et al., 2002), which are associated with aberrant localization of heterochromatin associated protein 1-alpha (Renella et al., 2011). The precise function of CDAN1 in erythroid cells is not well understood but it likely has a role in histone homeostasis during DNA replication (Ask et al., 2012). Intriguingly, CDAN1 has been shown to interact with SEC23B, (Renella et al., 2011), the gene most commonly mutated in CDA type II, (Schwarz et al., 2009; Iolascon et al., 2010), suggesting these mutations my disrupt a common pathway.

Acquired mutations in GATA1 and other erythroid transcription factors and epigenetic regulators can also lead to hematopoietic disorders. Intriguingly, the GATA1s mutation is commonly acquired in infants with Down Syndrome, and results in transient myeloproliferative syndrome (Wechsler et al., 2002), highlighting the context dependent nature of this mutation. Accumulation of cooperating mutations, most notably in CTCF and members of the cohesion complex, results in progression of TAM progression to Myeloid Leukemia of Down Syndrome (Yoshida et al., 2013). Myelodysplastic syndromes are a group of heterogeneous clonal disorders of hematopoietic stem cells characterized by bone marrow failure, ineffective red blood cell production, and increased risk of Acute Myeloid Leukemia (AML) (Dotson and Lebowicz 2021). MDS is associated with mutations in a wide variety epigenetic regulators and spicing factors, (Sperling et al., 2017), and disruption of the epigenetic landscape (Bond et al., 2020). Impaired upregulation of GATA1 due to alterations in the epigenetic landscape have been implicated in the dyserythropoiesis associated with MDS (Fadilah et al., 2002; Hopfer et al., 2012). The dyserythropoiesis associated with MDS is also associated with failure of nuclear and chromatin condensation, and disruptions in developmentally conserved changes in nuclear envelope and histone dynamics (Zhao et al., 2016a; Zhao et al., 2019).

## Conclusion

The epigenetic and transcriptional control of terminal erythroid maturation is complex, with each stage of erythropoiesis tightly regulated by various interdependent epigenetic mechanisms. Mutations that disrupt the transcriptional and epigenetic control of erythropoiesis lead to inherited anemias, highlighting the critical nature of these interactions. Understanding the fundamental mechanisms that govern erythroid maturation is essential to understanding the how mutations or other genetic perturbations result in disordered erythropoiesis, and to designing rational therapies.

## Data Availability

The original contributions presented in the study are included in the article/Supplementary Material, further inquiries can be directed to the corresponding author.
